# A potent synthetic nanobody with broad-spectrum activity neutralizes SARS-CoV-2 virus and the Omicron variant BA.1 through a unique binding mode

**DOI:** 10.1186/s12951-022-01619-y

**Published:** 2022-09-15

**Authors:** Dongping Zhao, Liqin Liu, Xinlin Liu, Jinlei Zhang, Yuqing Yin, Linli Luan, Dingwen Jiang, Xiong Yang, Lei Li, Hualong Xiong, Dongming Xing, Qingbing Zheng, Ningshao Xia, Yuyong Tao, Shaowei Li, Haiming Huang

**Affiliations:** 1 Qingdao Cancer Institute, The Affiliated Hospital of Qingdao University, Qingdao, 266071 China; 2 Noventi Biopharmaceuticals Co., Ltd, Shanghai, 201203 China; 3 State Key Laboratory of Molecular Vaccinology and Molecular Diagnostics, School of Public Health, School of Life Sciences, Xiamen University, Xiamen, 361102 China; 4 National Institute of Diagnostics and Vaccine Development in Infectious Diseases, Xiamen University, Xiamen, 361102 China; 5 Guangxi Asia United Antibody Medical Co., Ltd, Hezhou, 542899 Guangxi China; 6 Department of Clinical Laboratory, Ministry of Education Key Laboratory for Membraneless Organelles & Cellular Dynamics, Biomedical Sciences and Health Laboratory of Anhui Province, School of Life Sciences, Division of Life Sciences and Medicine, The First Affiliated Hospital of USTC, University of Science and Technology of China, 230027 Hefei, People’s Republic of China

**Keywords:** SARS-CoV-2, Neutralizing antibody, Synthetic nanobody, Broad-spectrum, Omicron, Cryo-EM

## Abstract

The major challenge to controlling the COVID pandemic is the rapid mutation rate of the SARS-CoV-2 virus, leading to the escape of the protection of vaccines and most of the neutralizing antibodies to date. Thus, it is essential to develop neutralizing antibodies with broad-spectrum activity targeting multiple SARS-CoV-2 variants. Here, we report a synthetic nanobody (named C5G2) obtained by phage display and subsequent antibody engineering. C5G2 has a single-digit nanomolar binding affinity to the RBD domain and inhibits its binding to ACE2 with an IC_50_ of 3.7 nM. Pseudovirus assays indicated that monovalent C5G2 could protect the cells from infection with SARS-CoV-2 wild-type virus and most of the viruses of concern, i.e., Alpha, Beta, Gamma and Omicron variants. Strikingly, C5G2 has the highest potency against Omicron BA.1 among all the variants, with an IC_50_ of 4.9 ng/mL. The cryo-EM structure of C5G2 in complex with the spike trimer showed that C5G2 binds to RBD mainly through its CDR3 at a conserved region that does not overlap with the ACE2 binding surface. Additionally, C5G2 binds simultaneously to the neighboring NTD domain of the spike trimer through the same CDR3 loop, which may further increase its potency against viral infection. Third, the steric hindrance caused by FR2 of C5G2 could inhibit the binding of ACE2 to RBD as well. Thus, this triple-function nanobody may serve as an effective drug for prophylaxis and therapy against Omicron as well as future variants.

**Supplementary Information:**

The online version contains supplementary material available at 10.1186/s12951-022-01619-y.

## Introduction

COVID-19 (coronavirus disease 2019) has become a pandemic worldwide since the end of 2019. As of August 8, 2022, it has caused more than 580 million infection cases and more than 6 million deaths (https://covid19.who.int/). SARS-CoV-2, a virus belonging to the beta coronavirus family, causes the disease [1, 2]. SARS-CoV-2 enters human cells via its spike protein, which is capable of binding to ACE2 (angiotensin-converting enzyme 2) receptors on the surface of host cells [3, 4]. The spike protein is composed of the S1 subunit at its N-terminus and the S2 subunit at its C-terminus [5]. S1 contains an N-terminal domain (NTD) and a receptor-binding domain (RBD) that engages the binding to ACE2 receptors on host cells [5–7]. Upon binding, the S2 subunit mediates membrane fusion between the viral and host cells to complete the viral entry [8, 9]. Based on this biological process, the spike protein has been the target for neutralizing antibody therapy and vaccine protection [10–12]. However, the rapid mutation rate of SARS-CoV-2 challenges the development of neutralizing antibodies and vaccines.

There are two groups of neutralizing antibodies targeting the spike protein of SARS-CoV-2, the majority of which are those binding to RBD, thus blocking virus interaction with ACE2[10, 11, 13]. In addition, antibodies targeting the NTD could also have neutralization capacity [14, 15]. The mechanism of anti-RBD neutralizing antibodies can also be classified into two groups [16]. The first group of antibodies bind to RBD and block its interaction with ACE2 directly, thus protecting the host cells from virus infection [10, 17, 18]. In contrast, the other group of antibodies interacts with RBD at epitopes distinct from the binding interface between RBD and ACE2, resulting in virus entry prevention as well, due to partially, if not completely, the conformational changes of RBD [19, 20]. This type of neutralizing antibody is more likely to act as a broad-spectrum antibody, as it binds to conserved residues in the RBD/NTD across SARS-CoV-2 variants.

The neutralizing antibodies were mainly derived from B cells of COVID-19 patients and immunized animals [21, 22] or phage-displayed antibody libraries, either of natural origin or synthetic ones [10, 11, 17]. Synthetic antibody library technology harnesses the frameworks of natural antibodies, based on which only the CDRs (complementarity-determining regions) are partially or completely randomized to form large content (diversity usually greater than 10^9^ pfu) libraries [23–25]. Although independent of the immune system, the notion of the synthetic antibody as therapeutic has been proven feasible and practical, as exemplified by the MorphoSys antibody drug guselkumab. Guselkumab, targeting IL23, was developed from the MorphoSys synthetic library HuCAL and approved to treat plaque psoriasis by the FDA in 2017 [26, 27]. The HuCAL library was constructed using human germlines as scaffolds [28]. Since then, many different antibody scaffolds have been employed to generate synthetic antibody libraries, including nanobodies. Nanobodies or single domain antibodies are the variable regions of heavy chain-only antibodies from the camelid immune system [29, 30]. One of the benefits of using nanobodies to construct synthetic antibody libraries is that a nanobody has only three CDRs (*versus* six CDRs in a conventional antibody), which makes the mutation process easier than those of using conventional IgG (Fab or scFv fragment) as scaffolds [31]. Many nanobodies, either natural [11, 32] or synthetic ones [10, 33], have been reported to have neutralizing activity against the SARS-CoV-2 virus. Unfortunately, there are few nanobodies reported to date that have broad-spectrum neutralizing activities against multiple SARS-CoV-2 variants, especially the newly emerged Omicron.

We first described in this study the construction of a synthetic nanobody library based on a camel nanobody by a novel randomization strategy. We selected this library against the SARS-CoV-2 spike protein, S1 subunit and RBD domain by phage display and obtained seven nanobodies in total. One of the RBD-binding nanobodies, named C5, is capable of competing with ACE2 to bind to RBD. Protein engineering technology was applied to improve the binding affinity of C5 to RBD and its solubility and yield. The engineered variant, i.e., C5G2 has proven its potency to protect living cells from infection by SARS-CoV-2 pseudovirus. In addition, C5G2 could inhibit multiple variants, including Omicron, infecting living cells with high potency in pseudovirus assays. We further solved the cryo-EM structure of C5G2 in complex with the spike protein trimer, uncovering the structural basis of the broad-spectrum neutralizing activity.

## Results

### The design, construction and quality control of a synthetic nanobody library

**Fig. 1:**
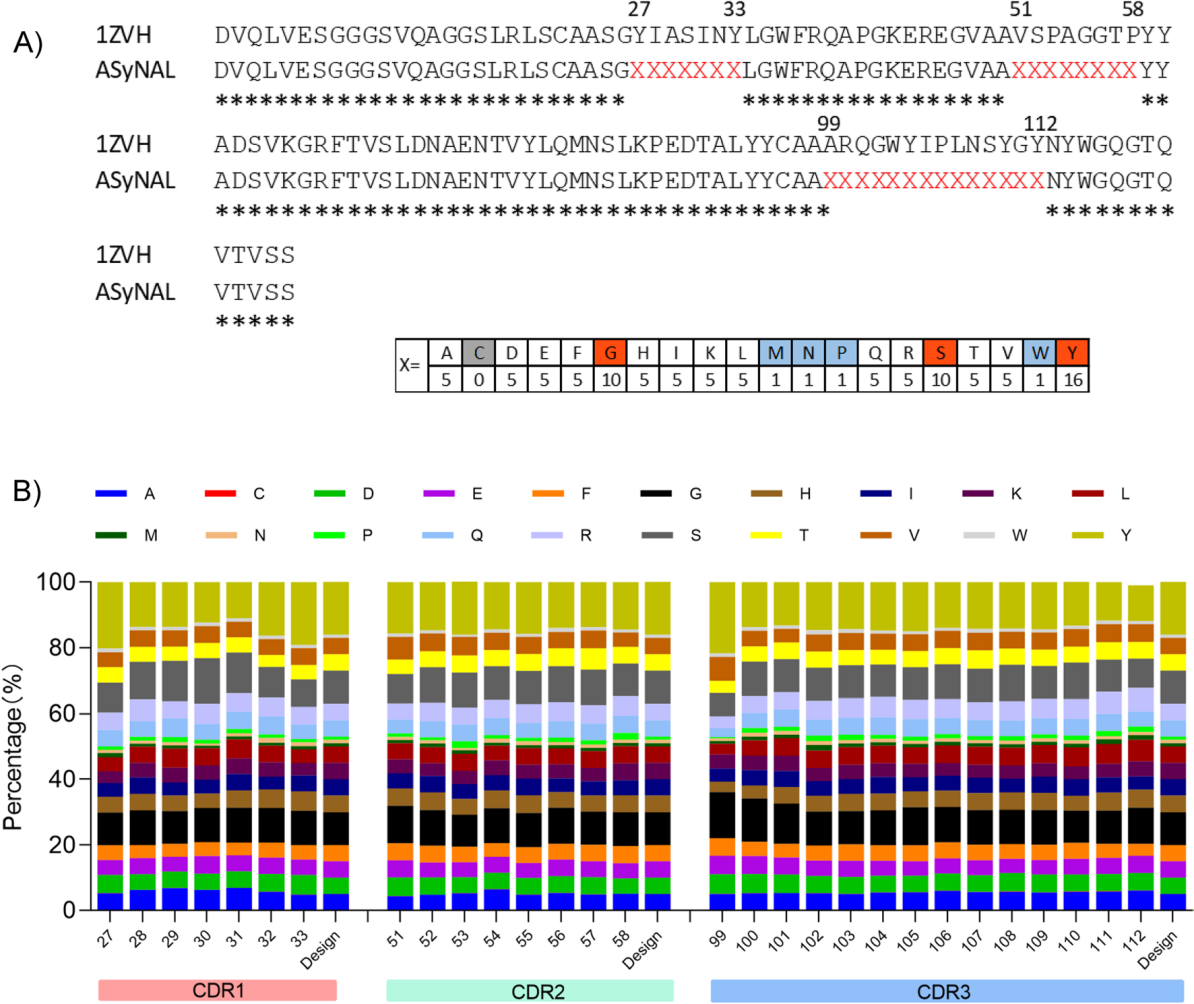
Construction and quality control of a synthetic nanobody library **A**. Sequence alignment of a camel nanobody (1ZVH) with the ASyNAL scaffold, showing the randomization regions in CDR1(27–33), CDR2(51–58) and CDR3(99–112) by X, respectively. The X is a mix of amino acids with the ratio shown below. **B**. NGS data (n = 20,112) showing the amino acid occurrence rate of ASyNAL at each position *vs* the designed rate. A total of 20,112 nanobody sequences were aligned, the amino acid residues at CDR regions were retrieved, and the rate of each amino acid occurrence (%) at each position was calculated

To obtain antibodies for diverse antigens by phage display, we first sought to construct a synthetic nanobody library. To this end, a natural nanobody (PDB no. 1ZVH) [34], which recognizes lysozyme, was selected as the template for library construction (Fig. 1A). In our library design, residues 27 to 33 of complementarity-determining region 1 (CDR1), 51 to 58 of CDR2 and 99 to 112 of CDR3 were randomized simultaneously (Fig. 1A). The length of each CDR in the library was kept the same as that of the template. At each position of the CDRs, an amino acid mix was incorporated by the Kunkel method [35, 36]. The amino acid mix (X) is composed of 19 natural amino acids, excluding cysteine (Fig. 1A), to prevent the formation of unexpected disulfide bonds, although extra interloop disulfide bonds are very common in naturally occurring nanobodies, especially camel-derived nanobodies [29]. The details of the construction of the library are described in the Materials & Methods section. The resultant diversity of the library was ~ 3 × 10^9^ cfu(colony-forming unit). The DNA deep sequencing results indicated that the mutated molecules had the expected amino acid percentage as designed at each CDR (Fig. 1B). This library was then named the AUAM synthetic nanobody library (ASyNAL).

We then panned the library against seven diverse antigens by phage display. Other than lysozyme, those antigens were cytoplasmic proteins, i.e., SH2 domain of tyrosine-protein kinase Fyn (Fyn_SH2) and glutathione S-transferase (GST); the extracellular domain of cell membrane proteins, i.e. human epidermal growth factor receptor 2 (Her2), Siglec15 and virus proteins, i.e., human SARS-CoV-2 spike protein and porcine circovirus (PCV) 2d protein. As shown in Table 1, we were able to obtain at least one nanobody for each of those seven antigens verified by phage ELISA, indicating the versatile applications of ASyNAL in the generation of nanobodies for diverse antigens.

**Table 1:** Antigens and their nanobodies selected from ASyNAL libra

Antigen	Unique Nb	Category
Lysozyme	10	Secreted enzyme
Fyn SH2	1	Cytoplasmic protein
GST	6	Cytoplasmic protein
Her2	9	Extracellular domain of membrane
Siglec 15	11	Extracellular domain of membrane
SARS-CoV-2 spike	7	Virus protein
Swine Pcv-2d	8	Virus protein

### Characterization of potential neutralizing nanobodies against the SARS-CoV-2 spike protein

**Fig. 2:**
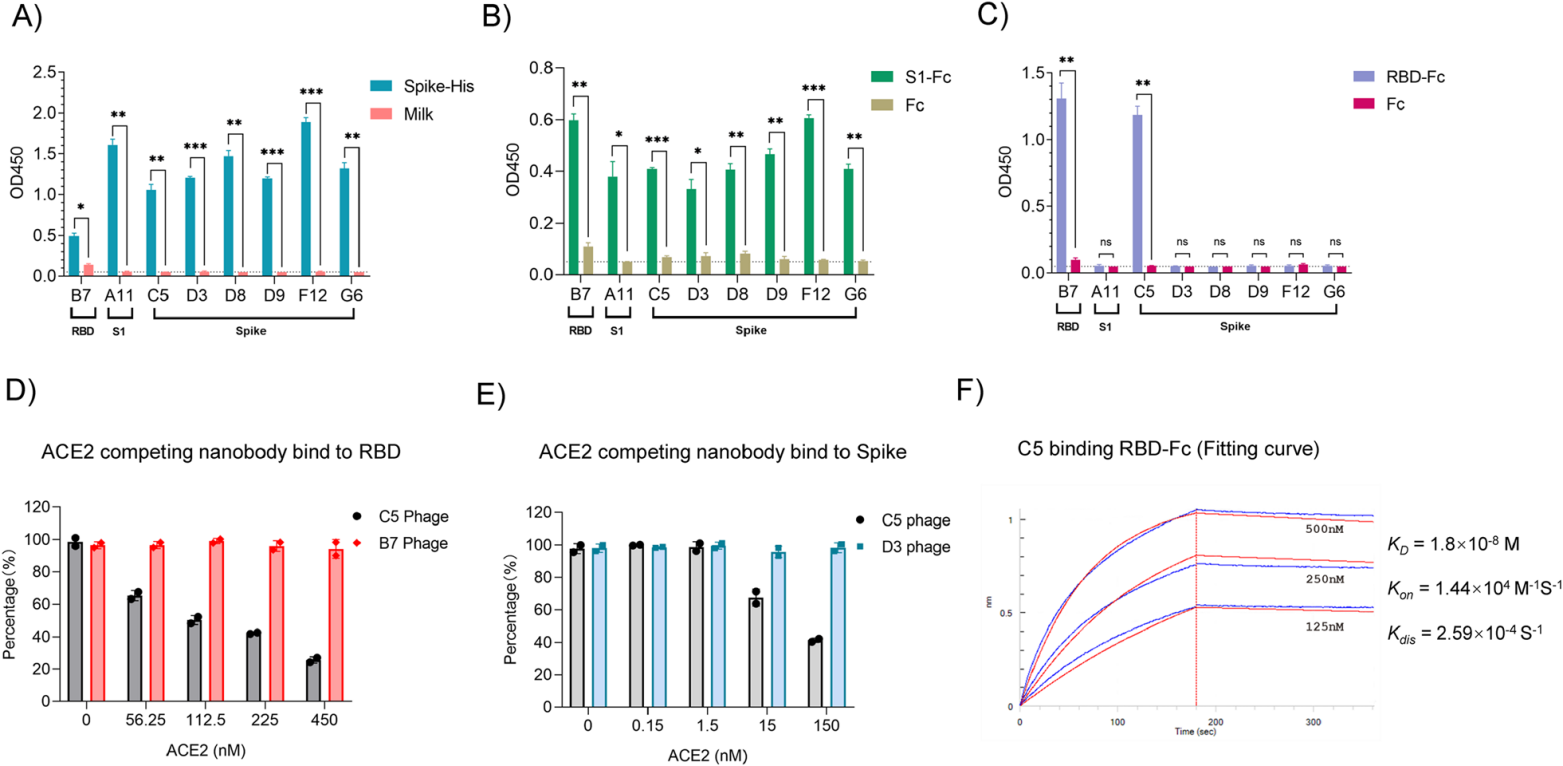
Identification of the C5 clone as a spike protein/RBD binder and competing with both Spike and RBD binding to ACE2 Phage panning of the ASyNAL library against antigens RBD, S1 and spike protein respectively generated eight nanobody clones. Clone B7 is for RBD, A11 is for S1 and C5, D3, D8, D9, F12, and G6 are for spike protein. The cross-reactivities of the eight clones to Spike **A**, S1 **B** and RBD **C** were determined by phage ELISA. Milk or Fc were applied as negative control in ELISA. Two replicates were performed, and the p-value was performed with Student’s t-test (ns, no significant; *p < 0.05; **p < 0.01; ***p < 0.001). Competitive ELISA showed that C5 phage and ACE2 competed with each other to bind RBD **D** and the full-length spike protein **E**. As controls, B7 phage and D3 phage had no competition. **F** Binding curve of C5 protein to RBD in BLI assay. The affinity is 18 nM

In an attempt to obtain neutralizing nanobodies against SARS-CoV-2, we panned the ASyNAL library against three antigens, including the spike protein, S1 domain and RBD. As a result, we successfully obtained six spike-specific nanobodies (named C5, D3, D8, D9, F12 and G6), one S1-specific nanobody (A11) and one RBD-specific nanobody (B7) as identified by phage ELISA (Fig. 2A–C, Additional file 1: Table S1, Fig. S1, milk and Fc were applied as negative controls). DNA sequencing showed that the D3 clone (from spike panning) and A11 (from S1 panning) are sequentially identical. Thus, we obtained seven unique nanobodies for the spike protein. To map the binding epitopes of those nanobodies, we then applied a cross-reactive phage ELISA to check the binding of those seven nanobodies to the spike protein and its fragments. All seven nanobodies bound the spike protein as expected (Fig. 2A). Furthermore, they all bound to S1 as well (Fig. 2B), but only nanobodies B7 and C5 could bind to the RBD (Fig. 2C), implying that the other five clones are likely NTD binders (Additional file 1: Figure S1A). In a competitive ELISA, NTD-binding nanobodies D8, D9, F12 and G6, but not RBD binder B7, inhibited D3 phage binding to the spike protein (Additional file 1: Figure S1B), showing that they may share the same (or overlapping) epitope as the D3 clone in the NTD domain (Additional file 1: Figure S1A & Table S1).

The binding affinities of these nanobodies to their screening antigens (Spike, S1 or RBD) were measured by biolayer interferometry (BLI). As shown in Table 2, the binding affinities (KD) range from 10^–10^ M to 10^–7^ M, which are similar to those selected from synthetic nanobody libraries [10] as well as natural nanobody libraries [11]. Furthermore, we investigated the size exclusion chromatography (SEC) profiles of the nanobodies, and the results indicated that, other than C5, which has aggregation (data not shown), five nanobodies (B7, D8, D9, F12, G6) showed one single peak, and nanobodies A11 and D3 showed minimal (~ 5%) aggregation (Additional file 1: Figure S2).

**Table 2:** Anti-Spike nanobody affinity and neutralization potency

Antigen		B7	A11/D3	D8	D9	F12	G6	C5	C5G2	mNb6
		RBD	S1/Spike	Spike	Spike	Spike	Spike	Spike	Spike	Spike
Spike binding	KD(M)			3.05E-08	2.61E-07	1.46E-07	2.48E-07			
	Kon(1/Ms)			7.71E + 04	1.87E + 05	2.51E + 05	1.40E + 05			
	Kdis(1/s)			2.53E-03	4.87E-02	3.66E-02	3.46E-02			
S1 binding	KD(M)		5.58E-10							
	Kon(1/Ms)		8.83E + 05							
	Kdis(1/s)		4.93E-04							
RBD binding	KD(M)	2.66E-07						1.80E-08	1.62E-09	3.48E-09
	Kon(1/Ms)	2.59E + 05						1.44E + 04	1.76E + 04	1.63 + 04
	Kdis(1/s)	6.87E-02						2.59E-04	2.84E-05	5.66E-05
Pseudo- virus IC_50_(M)	WT		N.E.					N.E.	1.59E-09	1.37E-08
	Alpha		N.E.					N.E.	9.5E-10	2.69E-08
	Beta		N.E.					8.16E-08	5.6E-10	N.E.
	Gamma		N.E.					N.E.	1.06E-09	N.E.
	Delta		N.E.					N.E.	N.E.	2.05E-08
	Omicron		N.E.					2.1E-09	3.0E-10	N.E.

NE.   no efficacy

As the spike protein of SARS-CoV-2 binds to human angiotensin-converting enzyme 2 (ACE2) through its RBD domain to initiate the entry of the virus into human cells, we wondered if the RBD-specific nanobodies B7 and C5 identified in this study could interfere with the binding of ACE2 to the RBD. Unfortunately, the yield of C5 protein was very poor when expressed in the bacterial host BL21. Therefore, we were unable to use the C5 protein as a competitor to interfere with the binding of the spike protein to ACE2 by competitive ELISA. In contrast, we tried to use ACE2 to interfere with the binding of C5/B7 nanobodies displayed on phage to S protein and RBD (i.e., phage competitive ELISA). As shown in Fig. 2D, the binding of C5, but not B7, to RBD could be inhibited when using ACE2 protein as the competitor. Furthermore, the binding of C5 to the spike protein could also be blocked by the ACE2 protein (Fig. 2E). Based on the data, we deduced that C5 could be used as a blocker against spike binding to ACE2 to neutralize the virus.

Unfortunately, C5 showed no neutralizing activity against SARS-CoV-2 pseudovirus, as the IC_50_ was greater than 1000 ng/ml (Fig. 4B), probably due to its low binding affinity to RBD (1.80 × 10^–8^ M) (Fig. 2F). In addition, as the protein yield and SEC profile were poor, we sought to engineer C5 by phage display to further improve its affinity and hopefully biophysical properties.

### Affinity maturation of the C5 nanobody by phage display

**Fig. 3:**
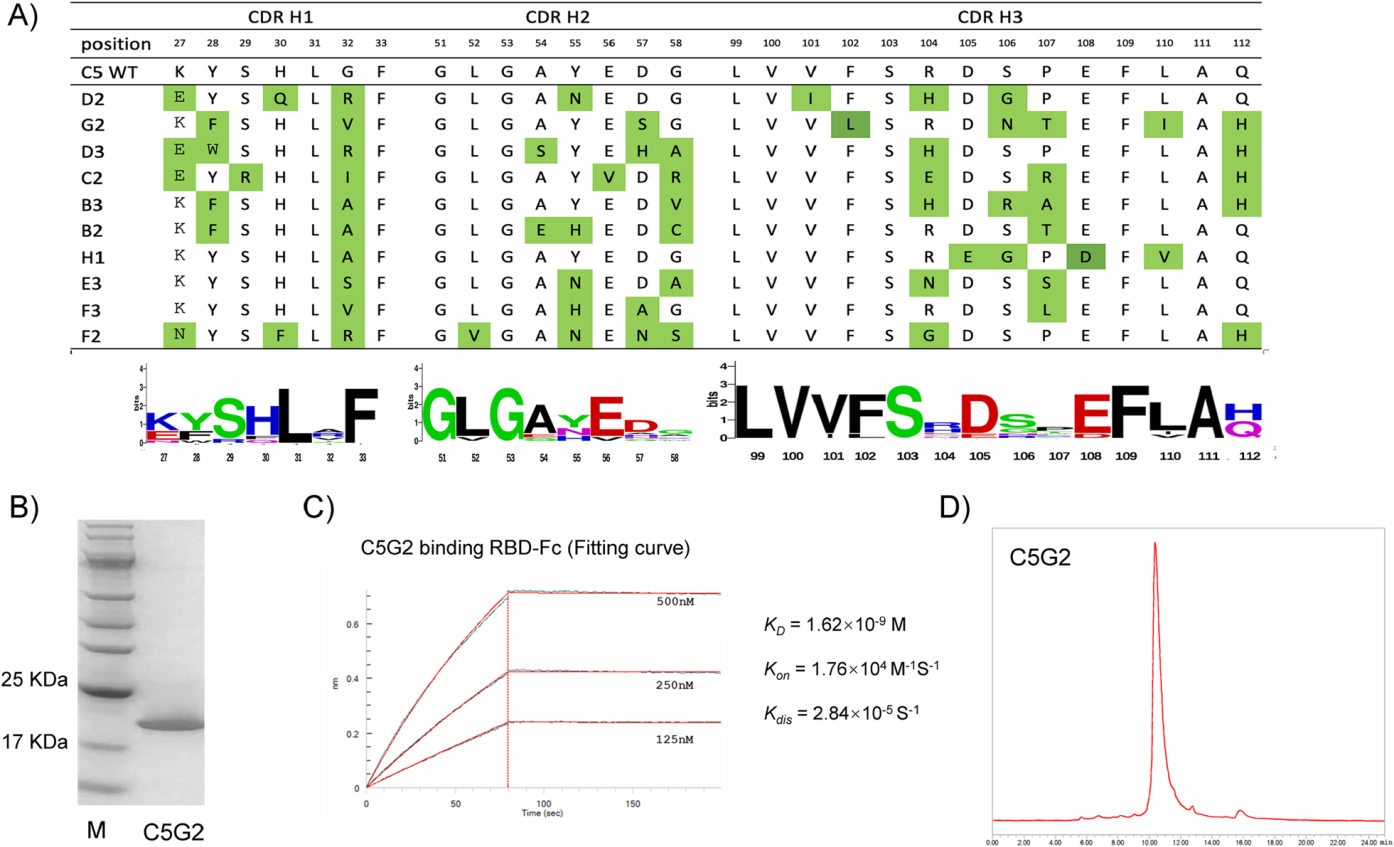
Affinity maturation of C5 clone by phage display led to the identification of the C5G2 clone **A** The C5 clone was used as the template to generate an optimization library with mutations at all CDRs. Panning of this library against RBD resulted in ten C5 variants. The CDR regions of these ten clones were aligned. The alignments represented by Weblogo are shown underneath each CDR region. **B** The C5G2 variant was purified from the E. coli host and resolved by SDS‒PAGE. **C** Binding curve of the C5G2 protein to RBD in the BLI assay. The binding affinity increased from 18 nM to 1.62 nM. **D** Size exclusion chromatography (SEC) profile of C5G2 protein resolved in a Tosho TSKgel G3000SW column

To increase the binding affinity of nanobody C5 to RBD, we applied a method called “soft randomization” [37]. All three CDRs of C5 were randomized simultaneously by doped oligos using the Kunkel method, resulting in a biased library (see Methods). After four rounds of phage panning, ten C5 variants that specifically bind to RBD were obtained, and their three CDRs were multi-aligned (Fig. 3A). The multi-alignment results clearly showed the variable residues and the conserved residues in the three CDRs. Notably, the variable residues of CDR3 gathered at the end of the loop, i.e., residues 104, 106 and 107. This highly variable region might enable CDR3 to evolve extra binding capacity other than the regions at the central part.

As the C5 protein was highly insoluble (data not shown), we expected a high yield of protein from those ten C5 variants. Fortunately, we were able to purify variant G2 (hereafter referred to as C5G2) with a significant increase in yield, i.e., from 0.3 mg to 5 mg protein per liter culture. The purified C5G2 was above 95% purity as resolved by SDS‒PAGE (Fig. 3B). The binding affinity of C5G2 to RBD (1.62 × 10^–9^ M) is ~ tenfold higher than that of the original C5 (Fig. 3C), mainly due to the decreased K_*dis*_ rate, which is from 2.59 × 10^–4^ s^−1^ to 2.84 × 10^–5^ s^−1^ (Table 2). The single peak in the SEC profile of C5G2 also indicated that there was no aggregation in this C5 optimized variant (Fig. 3D).

### C5G2 showed broad-spectrum neutralizing activity against SARS-CoV-2 variants in pseudovirus assays

**Fig. 4:**
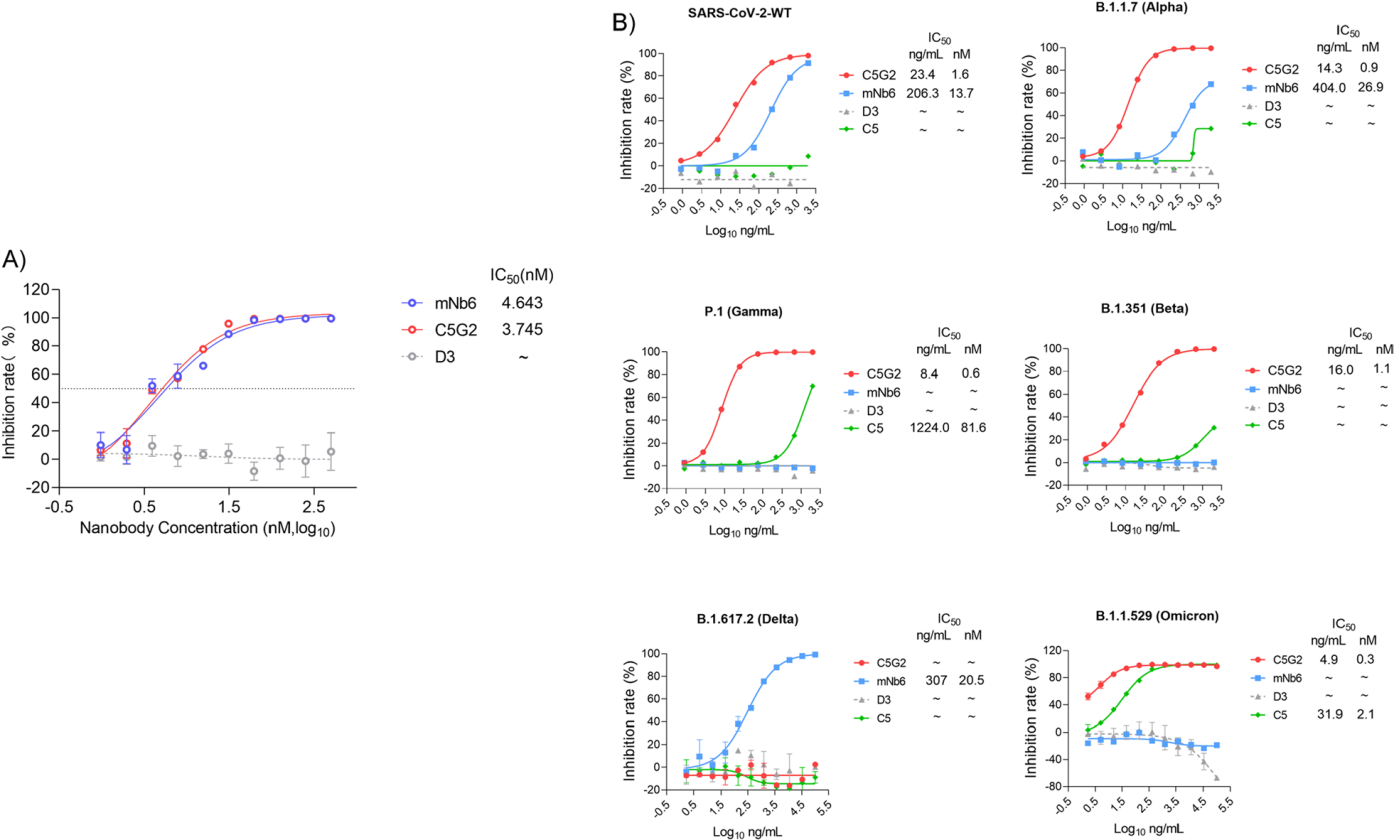
Pseudovirus assay of the C5G2 nanobody **A** Competitive ELISA showed that the C5G2 protein, but not D3, competed with ACE2 to bind RBD with an IC50 of 3.75 nM. As a control, the IC50 of mNb6 for the competition was 4.64 nM. The experiment was performed in triplicate. **B** Pseudovirus assay of C5G2’s inhibition potency to protect cells from virus infection. The wild-type virus and Alpha, Beta Gamma, Delta and Omicron (BA.1) viruses were tested. mNb6 was used as a positive control. Nanobody D3 binding to NTD of S1 was the negative control. The IC_50_ values are shown both in ng/ml and nM (nmol/L)

We then tested whether affinity-matured C5G2 could inhibit the binding of RBD to ACE2 and subsequently neutralize SARS-CoV-2 pseudovirus infection. We also included the D3 nanobody in this study as the negative control and the previously reported nanobody mNb6 [10] as the positive control. We first applied a competitive ELISA, in which C5G2 protein and the control proteins were employed to compete with ACE2 binding to RBD. As shown in Fig. 4A, with the increase in C5G2 protein concentration, the binding of RBD to ACE2 decreased, and the measured IC_50_ was 3.75 nM, which was comparable to that of mNb6 (4.64 nM). Meanwhile, the controlled D3 protein, which recognizes a non-RBD epitope, showed no inhibition at all. Based on the data, the C5G2 nanobody could interfere with the binding of RBD to ACE2, and the efficiency was close to that of the reported potent nanobody mNb6.

We then applied a pseudovirus assay to test whether the C5G2 protein could inhibit the infection of living cells by pseudoviruses. As shown in Fig. 4B, C5G2 could neutralize the WT pseudovirus with high potency, as the IC_50_ was 1.56 nM. Meanwhile, the IC_50_ of mNb6 is 13.7 nM. C5G2 is approximately ninefold more potent than mNb6 in this experiment. Surprisingly, C5G2 showed even high potency in neutralizing alpha, beta and gamma and Omicron(BA.1) variants, with IC_50_ of 0.95 nM, 1.06 nM, 0.56 nM and 0.3 nM, respectively (Fig. 4B). Unfortunately, C5G2 showed no neutralizing efficacy against the Delta variant. Taken together, C5G2 showed broad-spectrum neutralizing activities against most VOCs except for the Delta variant.

### Structural basis for the broad-spectrum neutralization activity of C5G2

**Fig. 5:**
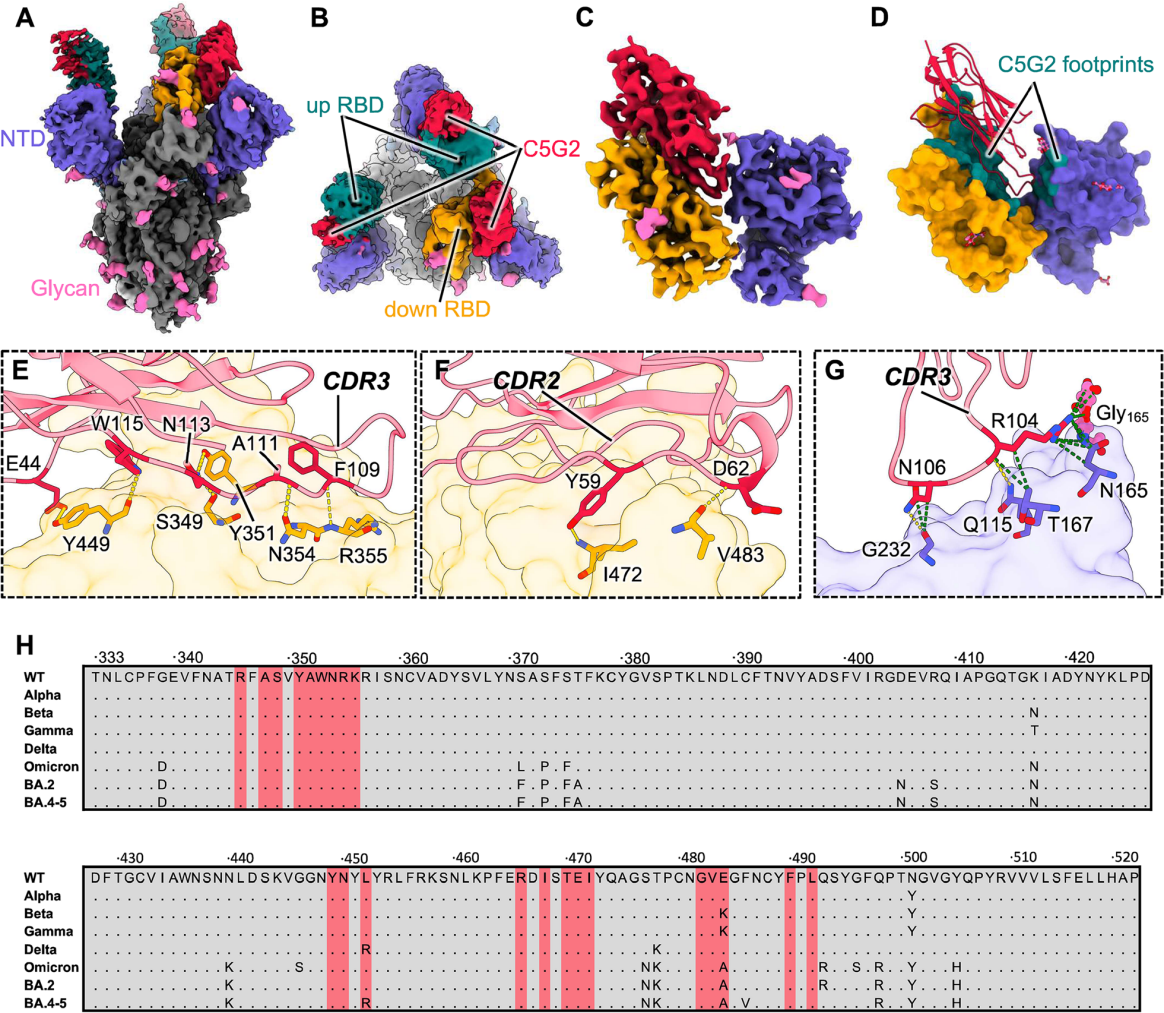
Structural basis for the broad neutralization of C5G2. **A**, **B** Cryo-EM density maps of SARS-CoV-2 spike protein in complex with C5G2 viewed from the side **A** and top **B**, respectively. C5G2 is colored red, and the up RBD, down RBD, NTD, and glycans of the spike are colored teal, orange, blue and pink, respectively. The rest density of the spike is colored gray. **C** The 3.88 Å density map of local refinement of C5G2 and bound RBD and NTD. **D** Atomic model of C5G2:RBD:NTD. C5G2 is presented as a cartoon, and RBD and NTD are presented as surface representations. The epitopes of C5G2 on the RBD and NTD are both colored in teal. The decorated glycans on the RBD and NTD are shown as pink sticks. **E**, **G** Details of the interaction between C5G2 and the RBD **E**, **F** and NTD **G**. C5G2 is shown as a transparent cartoon, and the RBD and NTD are shown as a transparent surface. The residues involved in the hydrogen bond (gold dotted lines) interaction are shown as sticks. The contacts between C5G2 and NTD are also presented as green dotted lines. **H** RBD sequences of SARS-CoV-2 WT and its seven VOC variants (Alpha, Beta, Gamma, Delta, Omicron and its sublineages BA.2, BA.4/5) with a highlighted footprint of C5G2 (colored in light red)

To decipher the molecular basis for the neutralization breadth of C5G2, we determined the cryo-electron microscopy (cryo-EM) structure of C5G2 in complex with SARS-CoV-2 S protein at an overall resolution of 3.57 Å (Fig. 5A, B, Additional file 1: Fig. S3 and Table S2). Compared to the wildly reported structure of the prefusion spike, which has one RBD in the open (up) state and the other two in the closed (down) state, C5G2 could recognize both the opened and closed RBDs (Fig. 5B). Due to its relatively smaller size than conventional IgG antibody (or Fab) and its long CDR3 loop, C5G2 could insert itself into the slit between the RBD and the adjacent NTD and make contact with both the RBD and NTD (Fig. 5B). Unfortunately, because of the structural flexibility of the RBD, the interaction details were not revealed in the initial reconstruction. Subsequently, we further performed a local refinement of one C5G2 plus the surrounding RBD and NTD (C5G2: RBD: NTD) at a resolution of 3.88 Å (Fig. 5C, Additional file 1: Figure S3 and Table S2). Consequently, the high-resolution details of local refinement allowed us to build an atomic model of C5G2 and the bound RBD and NTD, which confirmed the simultaneous interaction of C5G2 with the RBD and NTD (Fig. 5D). C5G2 dominantly interacts with the RBD and results in an extremely large buried area of approximately 1,180 Å^2^ on the RBD. Meanwhile, the buried area of the same C5G2 on the NTD is approximately 200 Å^2^ (Fig. 5D).

C5G2 mainly uses its long CDR3 to mediate interactions with both the RBD and the NTD (Fig. 5E–G). First, the CDR3 residues F109, A111, N113 and W115 form 5 hydrogen bonds to the RBD (Fig. 5E). In addition, the CDR2 of C5G2 also interacts with the RBD and forms two additional hydrogen bonds mediated by residues Y59 and D62 (Fig. 5F). The framework 2(FR2) of C5G2 thus adopts an orientation that induces steric hindrance to the putative ACE2 that binds to the same RBD (Additional file 1: Figure S4). As a result, C5G2 showed strong inhibition potential against the binding of ACE2 to RBD (Fig. 4A). Furthermore, because of the long CDR3 loop, it extends to the neighboring NTD and forms 2 hydrogen bonds through residues R104 and N106 (Fig. 5G). Of note, the NTD residue N165 and decorated glycan both participate in the interaction with C5G2 (Fig. 5G). As the glycosylation of N165 functions as a switch of the up-to-down RBD conformation [38], this interaction may interfere with the function of the N165 glycan and subsequently enhance the inhibition of RBD binding to ACE2.

Since it is the RBD that mainly mediates the interaction with C5G2, we next explored the conservation of the C5G2 epitope on the RBD. A total of 22 residues (346, 348, 349, 351–356, 449, 450, 452, 466, 468, 470–472, 482–484, 490 and 492) on RBD are involved in the interactions with C5G2 (Fig. 5H). Of these, 91% (20 residues, except for 452 and 484) are highly conserved among most variants of interest (VOCs) of Alpha, Beta, Gamma, Delta, Omicron and its sublineages BA.2 and BA.4/5 (Fig. 5H). Although the epitope residue L452 was not involved in hydrogen bond or salt bridge interactions with C5G2, the L452R mutation on the Delta variant completely abolished the activity of C5G2 (Fig. 4B). Furthermore, although the previously dominant Omicron variant holds 15 unprecedented mutations in its RBD, C5G2 showed effective neutralization potency against Omicron pseudovirus (Fig. 4B). Structural analysis showed that of all the mutations contained in the Omicron sublineage, only L452R and E484A were located in the footprint of C5G2 (Fig. 5H), suggesting epitope conservation among SARS-CoV-2 and the Omicron variant.

## Discussion

A synthetic antibody library is a common and reliable way to obtain antibodies for diagnosis and therapy [10, 28, 39, 40]. In this study, we first constructed a universal synthetic nanobody library to generate nanobodies for diverse antigens. The library design strategy is straightforward and readily conducted. Although less sophisticated compared to the reported synthetic libraries [41, 42], the resulting library ASyNAL enabled us to obtain at least one nanobody for the randomly selected antigens (Table 1), proving its versatile utilization for nanobody generation. The binding affinity of the S protein (and its fragments) nanobodies from the ASyNAL library ranged from 5.58 × 10^–10^ M to 3.05 × 10^–7^ M (Table 2), which is within the affinity scope for synthetic and natural antibodies. The SEC profile of the S protein nanobodies indicated that most of the nanobodies have minimal aggregation. Therefore, the ASyNAL library is a reliable resource of nanobodies with high affinities and good biophysical properties. Furthermore, the NGS data from library QC and panning results may help to design more sophisticated libraries for both naive and engineered libraries (e.g., affinity maturation).

We were able to obtain nanobody C5G2 against the RBD of the SARS-CoV-2 spike protein by panning the ASyNAL library and subsequent antibody engineering. C5G2 binds to the S protein mainly through CDR2 and CDR3 on a very conserved RBD surface in both the up and down states (Fig. 5B, H). In addition, the long CDR3 extends itself down to the bottom of the slit formed by RBD and neighboring NTD and consequently makes contact with residues in NTD (Fig. 5D, G). This interaction may interfere with the function of the N165 glycan and may further lock the RBD in the down state. Furthermore, the FR2 of C5G2 sticks upward to the RBM (receptor binding motif) to cause steric hindrance to prevent potential ACE2 binding. This unique mechanism of C5G2 interaction with the S protein underlies its basis of the high potency of neutralization and broad activities (Fig. 4A&B).

According to Hastie et al., RBD antibodies can be classified into three groups: those that bind to the RBM, the outside face and the inner face [43]. The RBM binders, usually competing with ACE2 to bind to RBD, lose (or weaken) their binding and neutralizing potencies to the variants of the SARS-CoV-2 virus, especially the most recent Omicron [44]. For example, the ultrapotent nanobody mNb6 lost its activities to most of the VOCs (Fig. 4B), mainly due to its RBM binding property (Additional file 1: Figure S5). In contrast, the clinically approved Vir-S309 monoclonal antibody still neutralized Omicron with a slight potency decrease (VSV IC50 = 284 ng/mL) as it binds to a conserved and outer face of RBD (Additional file 1: Figure S5). The C5G2 nanobody in this study binds to an RBD epitope with > 90% residue conserved in the VOCs and 77.3% residues conserved in all the SARS-CoV-2 variants reported thus far (Additional file 1: Table S3), underlying its broad-spectrum activity against most of the VOCs (Fig. 4B). Moreover, we found that the C5G2 nanobody protected cells from swine coronavirus infection in a separate experiment (data not shown), implying its epitope conservation and broad activity across coronavirus species.

N165 is a conserved residue in the NTD, and its glycan plays a pivotal role in facilitating the transition of the RBD from the down state to the up state conformation and stabilizes the conformation by interacting with RBD residues Y351, T470, F490, and L452 [38, 45]. C5G2 can interact not only with the N165 glycan in the NTD (Fig. 5G) but also with all the residues (Y351, T470, F490 and L452) responsible for N165 glycan binding in the RBD (Fig. 5H). This interaction of C5G2 could block the N165 glycan-binding ability to RBD, as exemplified by neutralizing antibody 35B5 [45]and stabilize RBD in the down state, thus enhancing the inhibition efficiency of C5G2. ACE2 interacts with RBD in an up state during virus infection. However, the steric hindrance caused by FR2 of C5G2 upon binding could further inhibit ACE2 binding to the RBD. Therefore, C5G2 acts as a triple-function nanobody, i.e. RBD binder, NTD binder and ACE2 blocker (Additional file 1: Figure S4). As a result, monomeric C5G2 nanobodies inhibited most of the VOCs with high potency_,_ with IC50 values ranging from 4.9–23 ng/mL in the pseudovirus assay (Fig. 4B). Furthermore, the potency of C5G2 could be driven even higher by linking two or three nanobodies together, e.g., in the case of bimNb6 and tri-mNb6[10].

Unfortunately, the advantage of C5G2 for virus inhibition was compromised by the L452R mutation of RBD in the variant of Delta (Fig. 4B). Furthermore, the L452R mutation, also contained in the currently dominant variant BA.4/5(Fig. 5H), might diminish the activity of C5G2 as well. Linking C5G2 with a heterogeneous nanobody may compensate for this disadvantage, as exemplified by the bispecific sdAb bn03 (Additional file 1: Figure S5) [46]. Due to its renewable characteristics, further engineering of the CDR loops of C5G2 by directed evolution may also find a new nanobody variant to tolerate this mutation while maintaining the other functions. Taken together, this monomeric, small-size (15 kD) nanobody can compete with full IgG and bispecific nanobodies (Additional file 1: Figure S5, lower table) in preventing current SARS-CoV-2 and its variants through its unique mechanism of action. Due to the highly conserved epitopes in the RBD and NTD, C5G2 also holds promise for inhibiting emerging viruses in the future.

## Materials and methods

### Construction of a synthetic nanobody library

To construct a novel synthetic nanobody library, a natural nanobody (PDB no. 1ZVH) from the camel that recognizes lysozyme was used as a template for mutagenesis. The coding cDNA of 1ZVH was chemically synthesized and subcloned into a modified phage display vector of pComb3XSS, in which the amber stop codon (TAG) was mutated to CAG to facilitate nanobody display in *E. coli* without the supE gene, e.g., SS320 (Genentech). The 1ZVH construct was first transformed into *E. coli* SS320, and the phage was produced by adding helper phage M13KO7 (New England Biolabs). The successful display of 1ZVH was then verified by phage ELISA, in which the produced phage could be bound by immobilized lysozyme in a 96-well NUNC Maxisorp plate.

To increase the oligo incorporation efficiency, two ochre stop codons (TAAs) and one BamH I restriction enzyme site (GGATCC) in between were applied to replace the CDR3 region of 1ZVH to generate a so-called “stop template”. The “stop template” was transformed into *E. coli* CJ236 (New England Biolabs) to produce *du* single-strand DNA (*du*-ssDNA) as described previously [47]. The mutation strategy used degenerate oligo mix (X) to incorporate into the CDR1 (7 aa), CDR2 (8 aa) and CDR3 (14 aa) of the 1ZVH, respectively (Fig. 1A). The degenerate oligo mix X denotes 16% tyrosine, 10% glycine, 10% serine, 5% alanine, aspartate, glutamate, phenylalanine, histidine, isoleucine, lysine, leucine, glutamine, arginine, threonine, and valine, respectively, and 1% methionine, asparagine and tryptophan. The oligos for each CDR were synthesized by a TRIMER-like method by Genewiz (Suzhou, China). The CDR oligos were incorporated into the corresponding CDR regions simultaneously by standard Kunkel reaction as described previously [36]. The Kunkel product was transformed into 350 µL high-efficiency competent cell SS320 (with M13KO7 incorporated) made by electroporation as described previously [36]. The transformed SS320 were grown overnight in 2YT media supplemented with 50 μg/mL carbenicillin at 32 °C with a shaking speed of 200 rpm. To increase the incorporation percentage at the CDR3 region, the SS320 cells were pelleted, and the plasmid DNA was maxipreped. 20 µg plasmid DNA was digested by restriction enzyme BamH I to remove those DNA molecules with failed incorporation at the CDR3 region (i.e., the stop templates) for at least 4 h at 37 °C. The resultant DNA was purified in a PCR product purification column to remove ions and enzymes. The DNA sample was resuspended to a minimum volume, i.e., 60 µL H_2_O. The digested DNA sample was retransformed into 350 µL competent cell SS320 (+ M13KO7) by electroporation. The transformed SS320 cells were grown as above, and the phages in the supernatant were precipitated by PEG/NaCl (20%/2.5 M). The concentration of the phage solution was determined by serial dilution and by infection with XL1-Blue (Stratagene) as described previously [36].

### Library quality control by next-generation sequencing

The library DNA were applied as templates in PCR, and the nanobody-encoding sequences were amplified by Phanta Max Super-Fidelity DNA Polymerase (Vazyme). The PCR products were resolved in a 1.5% agarose gel, and the band was sliced for gel purification. The purified DNA was subjected to next-generation sequencing (NGS). More than 50 ng purified PCR fragments were used for library preparation. These PCR products were treated with End Prep Enzyme Mix for end repair, 5’ Phosphorylation and dA-tailing in one reaction, followed by a T-A ligation to add adaptors to both ends. Adaptor-ligated DNA was then performed using DNA Clean Beads. A second PCR was carried out with two primers carrying sequences that can anneal with flowcell to perform bridge PCR and index allowing for multiplexing. The final library product for sequencing was then purified by beads and qualified. The qualified libraries were sequenced paired-end 150 bp on an Illumina NovaSeq 6000. The method of data analysis was performed as described previously [36].

### Phage biopanning

Phage biopanning was performed as previously described [48]. Briefly, two wells of a 96-well microplate (NUNC) were coated with 0.5 μg antigen (e.g., RBD-Fc or other antigens) and 0.5 μg Fc in 100 μL 1 × PBS (137 mM NaCl, 3 mM KCl, 8 mM Na_2_HPO_4_ and 1.5 mM KH_2_PO_4_, pH = 7.6) overnight, respectively. The solution in the well was discarded, and 200 μL/well 2% skim milk was added for blocking at room temperature for 1 h. After incubation, the solution was discarded, and 100 μL/well phage-displayed nanobody library (~ 3.0 × 10^12^ phage clones, approximately 1000 × library diversity) was added to the Fc wells for preincubation to remove the nonspecific binders. After incubation at room temperature for 1 h, phage solution was then transferred into the RBD-Fc well for binding for 1 h. Nonbinding phages were washed away with PT buffer (1 × PBS + 0.05% Tween) at least 8 times. Bound phages were eluted with 100 mM HCl 100 μL/well. A 1/8 volume of Tris–HCl (1 M, pH = 11) was then added for neutralization. The half volume of the neutralized phage solution was then applied to infect a tenfold volume of actively growing *E. coli* XL1-Blue (Stratagene) for 30 min at 37 ℃. Then, M13KO7 helper phages (NEB, N0315S) were added at a final concentration of 1 × 10^10^ phages/mL for superinfection for 45 min. The XL1-blue culture was added to a 20-fold volume of 2YT medium (10 g yeast extract, 16 g tryptone, 5 g NaCl in 1 L water) supplemented with Carb (carbenicillin, 50 mg/μL) and Kana (kanamycin, 25 mg/μL) and grown overnight (14–16 h) at 32 °C and 200 rpm in a shaker. The overnight culture was centrifuged, and the supernatant was precipitated by 1/5 volume of PEG/NaCl (20% PEG 8000/2.5 M NaCl). The amplified phage in the pellets were resuspended in 1 mL 1 × PBS and applied as the input phage for the next round of panning. For C5 variant library panning, from the second round, the RBD-Fc antigen was decreased from 0.5 μg to 0.2 μg (2nd round), 0.1 μg (3rd round), and 0.05 μg (4th round) to increase the selection stringency.

### Phage ELISA

In a 96-well NUNC microplate, 0.1 μg/well RBD-Fc (or other antigens) and 0.1 μg/well Fc were coated in wells in 50 μL 1 × PBS. After incubation at 4 °C overnight, the solution in the well was discarded, and 100 μL/well 2% milk was added for blocking at room temperature for 1 h. Phage solution (2 × 50 μL) was added to the RBD-Fc wells and Fc wells for binding for 1 h. Non-binding phages were washed away 8 times with PT buffer. 50 μL anti-M13/HRP conjugate (Sino Biological) was added and incubated for 30 min. After washing with PT buffer, 50 μL of TMB substrate was added to develop color according to the manufacturer’s instructions. Then, 100 μL of 1.0 M H_3_PO_4_ were added to stop the reaction, and signals were read spectrophotometrically at 450 nm in a BioTek plate reader. The RBD-Fc and Fc read wells were recorded, and the RBD-Fc/Fc ratio was calculated.

### Competitive phage ELISA

Spike-trimer-His (Acrobiosystem)/RBD-Fc (0.2 µg/well) was coated in a microplate in 50 µL of 1 × PBS buffer at 4 °C overnight. Then, 100 μL/well 2% milk was incubated for 1 h for blocking. After washing 3 times, a serial phage solution was added and incubated with spike-trimer-His for 30 min. Anti-M13/HRP conjugate (1:8000 dilution) and TMB were used to amplify the signals and to develop color, respectively. After stopping the reaction via 1.0 M H_3_PO_4_, the OD 450 value was read by a microplate reader. After that, the sub-saturation (80% of maximal effect) concentration of the phage solution was used for competitive ELISA.

Spike-trimer-His/RBD-Fc (0.2 µg/well) was coated for competitive phage ELISA. After incubation with 100 µL/well 2% milk for 1 h and 3 washes, the sub-saturation concentration of the phage solution was mixed with a decreased concentration of ACE2-Fc (Novoprotein). The mixture of phage and ACE2-Fc was added to wells coated with Spike-trimer-His/RBD-Fc and incubated for 30 min. M13/HRP conjugate, TMB, 1.0 M H_3_PO_4,_ and a microplate reader was used for subsequent experiments as described above.

### C5 affinity maturation library construction

The sequence of C5 in phagemid pComb3XSS was used as a template for library construction. The C5 du-ssDNA used for the Kunkel reaction was made as described previously [47]. Primer 1 (AGCTGTGCAGCAAGTGGA*TAAGGATCCTAA*CTAGGCTGGTTTCGTCAA), Primer 2 (CGCGAAGGAGTTGCTGCA*TAAGGATCCTAA*TACTACGCCGATAGCGTG), and Primer 3 (CTGTACTATTGTGCGGCC*TAAGGATCCTAA*AACTACTGGGGCCAAGGC) were used in a combinatorial reaction by the Kunkel method to construct a “stop template”, in which all CDRs were incorporated with the BamH I restriction enzyme recognition site *GGATCC* and two stop codons *TAA*. Then, Primer 4 (AGCTGTGCAGCAAGTGGAN_1_N_1_N_1_N_2_N_1_N_2_N_1_N_4_N_3_N_3_N_1_N_2_N_3_N_2_N_4_N_4_N_4_N_2_N_2_N_2_N_2_CTAGGCTGGTTTCGTCAA),

Primer 5 (CGCGAAGGAGTTGCTGCAN_4_N_4_N_2_N_3_N_2_N_4_N_4_N_4_N_2_N_4_N_3_N_1_N_2_N_1_N_2_N_4_N_1_N_1_N_4_N_1_N_2_N_4_N_4_N_2_TACTACGCCGATAGCGTG) and Primer 6 (CTGTACTATTGTGCGGCCN_3_N_2_N_4_N_4_N_2_N_2_N_4_N_2_N_2_N_2_N_2_N_2_N_1_N_4_N_3_N_3_N_4_N_2_N_4_N_1_N_2_N_1_N_4_N_3_N_3_N_3_N_4_N_4_N_1_N_1_N_2_N_2_N_2_N_3_N_2_N_4_N_4_N_3_N_1_N_3_N_1_N_4_AACTACTGGGGCCAAGGC) were applied simultaneously in a Kunkel method to synthesize the heteroduplex double-strand DNA (dsDNA), in which the designed mutations at each position were incorporated into the “Stop template”. In the above primers, N_1_ is composed of a mixture of 85% A and 5% G, T and C; N_2_ represents a mixture of 85% T and 5% A, G and C; N_3_ is a mixture of 85% C and 5% A, T and G; and N_4_ contains 85% G and 5% T, A and C. dsDNA was further digested by the restriction enzyme BamH I to remove the unreacted template molecules before transformation into *E. coli* SS320 (preinfected by M13KO7) by electroporation. The transformation efficiency (library diversity) was calculated by bacterial serial dilution as described [48]. The resulting phage library was precipitated by PEG/NaCl (20% PEG 8000/2.5 M NaCl) as described [36].

### Nanobody expression and purification

The cDNA encoding the nanobodies in the pComb3XSS vector was PCR amplified and subcloned into vector pET22b to express 6 × His tagged proteins. The expression constructs were transformed into *E. coli* BL21 (DE3). Single colonies were picked and grown in 2YT/Carb medium at 37 ℃ to OD_600_ = 0.8. IPTG was added to a final concentration of 0.2 mM, and protein expression was induced at 18 ℃ overnight. The culture was centrifuged, and the pellets were treated as previously described [49]. Briefly, pellets were resuspended in lysis buffer (to make 100 mL lysis buffer: 98 mL HEPES/NaCl (50 mM/500 mM) buffer, 1 mL Triton X-100, 1 mL 100 × protease inhibitor cocktail, 10 μL benzonase, 5% glycerol, 100 mg lysozyme). The lysate and the overnight supernatant were heated at 60 °C for 30 min to denature partially folded nanobodies. Heat-treated lysate and supernatant were centrifuged again and subjected to antibody purification. Antibodies were purified using Ni–NTA agarose (Qiagen) according to the manufacturer’s manual. The eluted proteins were buffer exchanged into 1 × PBS by Amicon Ultra4 Centrifugal Filter Units (Millipore). The final concentrations of the proteins were determined by the BCA method. The purity of the nanobodies was resolved in 15% SDS‒PAGE gels.

### Competitive protein ELISA

In a 96-well NUNC microplate, 0.2 μg ACE2-His (Novoprotein) was coated per well in 50 μL 1 × PBS at 4 °C overnight. Then, 100 μL/well 2% milk was added for blocking for 1 h. Serial RBD-Fc at increasing concentrations (0 nM, 3.125 nM, 6.25 nM…100 nM, 200 nM) was added to 9 wells coated with ACE2-His. The wells were washed 5 times with PT buffer after incubation for 1 h at room temperature. 50 μL of anti-IgG-HRP conjugate (Shanghai Ruian) was added to each well and incubated for 30 min. After washing 8 times with PT buffer, 50 μL of TMB substrate was added to develop color for 2 min. Then, 100 μL of 1.0 M H_3_PO_4_ were added to stop the reaction, and signals were read spectrophotometrically at 450 nm in a plate reader. Standard variation values were calculated using a 3-parameter logistic regression fit using Prism Software (GraphPad). The concentration for 80% of maximal effect (the subsaturation concentration) was applied for the following competitive ELISA. ACE2-His (0.2 μg/well) was coated in a 96-well NUNC microplate. After incubation at 4 ℃ overnight, 100 μL/well 2% milk was used for blocking. Sub-saturation concentrations of RBD-Fc were mixed with serial nanobody-His at decreased concentrations (500 nM, 250 nM, 125 nM… 0 nM). The mixture was added to 12 wells coated with ACE2-His and incubated at room temperature for 30 min. After incubation with anti-IgG-HRP and washing with PT buffer, TMB was used to develop the color. The reaction was terminated with 1.0 M H_3_PO_4,_ and the OD_450_ value was read through a BioTek Microplate Reader. Each reaction was performed in triplicate, and the mean of the readout was used for IC_50_ calculation.

### Size-exclusion chromatography

The buffer of the nanobody was changed to 1 × PBS (pH = 7.4) by ultrafiltration discs (Merck, PLBC07610). 50 μL nanobody (1 mg/mL) was filtered through a 0.2 μm filter. After that, filtered samples were prepared for HPLC analysis. An HPLC instrument (Waters, ACQUITY UPLC H-Clas) was used for purity analysis of proteins. PBS and a gel permeation chromatography column (TSKgel G3000SW_XL_, Tosoh Bioscience, 0008541) were used as the mobile phase and the stationary phase, respectively. The pump was running for 5 min, mainly to eliminate air bubbles in the system, and then all exhaust valves were closed. The mobile phase was run at a fixed rate (1 mL/min) until the baseline was stable. Then, the operating parameters (flow rate: 1 mL/min, pressure: 3.3 MPa, temperature: 25 °C, detection: UV 280 nm) of the sample were set in the software (Waters, Empower3). After that, the sample was injected into the sampling valve, and the line was monitored by software. The sample was allowed to run for 20 min (more than 1.5✕column volume) before stopping.

### Biolayer interferometry (BLI) assay

BLI experiments were carried out using an Octet RED96 System (ForteBio). The measurements were performed using Ni–NTA biosensors (ForteBio). Nanobodies with a 6 × His tag were immobilized on the biosensor tip surface. All steps were performed at 30 °C with shaking at 1000 rpm in a black 96-well plate, with a working volume of 200 μL in each well.

RBD-Fc at different concentrations (250 nM, 125 nM, 0 nM) in running buffer (1 × PBS + 0.5% BSA + 0.05% Tween) was applied for association and dissociation. The response data were normalized using Octet data analysis software version 9.0.0.14 (ForteBio).

### Pseudovirus-based neutralization assay

Vesicular stomatitis virus (VSV) dG-SARS-Cov2 virus was used as a pseudovirus, and BHK21-hACE2 was prepared for the neutralization assay [50]. Gradient diluted nanobodies were added to the VSV dG-SARS-Cov2 virus (MOI = 0.05) and then incubated at 37 °C for 1 h. All samples and viruses were diluted with 10% FBS-DMEM. After incubation, the mixture was added to BHK21-hACE2 and incubated for 12 h. Fluorescence images were obtained with Opera Phenix or Operetta CLS equipment (PerkinElmer) and analyzed by the Columbus system (PerkinElmer). The number of GFP-positive cells in the nanobody-treated group and in the nontreated group was compared to calculate the reduction ratio, which represents the neutralizing potency.

### Cryo-EM sample and data collection

Aliquots (3 μL) of 3.5 mg/mL mixtures of purified SARS-CoV-2 S protein (Acrobiosystem) in complex with excess C5G2 of three nAbs were incubated in 0.01% (v/v) digitonin (Sigma) and then loaded onto glow-discharged (60 s at 20 mA) holey carbon Quantifoil grids (R1.2/1.3, 200 mesh, Quantifoil Micro Tools) using a Vitrobot Mark IV (ThermoFisher Scientific) at 100% humidity and 4 °C. Data were acquired using SerialEM software on an FEI Tecnai F30 transmission electron microscope (ThermoFisher Scientific) operated at 300 kV and equipped with a Gatan K3 direct detector. Images were recorded in 36-frame movie mode at a nominal 39,000 × magnification in super-resolution mode with a pixel size of 0.389 Å. The total electron dose was set to 60 e^−^ Å^−2^, and the exposure time was 4.5 s.

### Image processing and 3D reconstruction

Drift and beam-induced motion correction were performed with MotionCor2 [51] to produce a micrograph from each movie. Contrast transfer function (CTF) fitting and phase-shift estimation were conducted with Gctf [52]. Micrographs with astigmatism, obvious drift, or contamination were discarded before reconstruction. The following reconstruction procedures were performed by using Cryosparc V3 [53]. In brief, particles were automatically picked by using the “Blob picker” or “Template picker”. Several rounds of reference-free 2D classifications were performed, and the selected good particles were then subjected to ab-initio reconstruction, heterogeneous refinement and final non-uniform refinement. The resolution of all density maps was determined by the gold-standard Fourier shell correlation curve, with a cutoff of 0.143 [54]. Local map resolution was estimated with ResMap [55].

### Atomic model building, refinement, and 3D visualization

The initial model of nAbs was generated from homology modelling by Accelrys Discovery Studio software (available from URL: https://www.3dsbiovia.com). The structure of the SARS-CoV-2 RBD from the structure of the WT trimeric spike (PDB no. 6VSB [56]) was used as the initial mode of our WT RBD. We initially fitted the template models into the corresponding final cryo-EM map using Chimera [57] and further corrected and adjusted them manually by real-space refinement in Coot [58]. The resulting models were then refined with Phenix.real_space_refine in PHENIX [59]. These operations were executed iteratively until the problematic regions, Ramachandran outliers, and poor rotamers were either eliminated or moved to favored regions. The final atomic models were validated with Molprobity [60, 61]. All figures were generated with Chimera or ChimeraX [62, 63].

### Data and code availability

Structure coordinates are deposited in the Protein Data Bank under accession codes 7XRP (RBD: NTD: C5G2). The corresponding EM density maps have been deposited in the Electron Microscopy Data Bank under accession numbers EMD-33416 (RBD: NTD: C5G2) and EMD-33415 (Spike: C5G2).

## Supplementary Information

**Additional file 1: Table S1. **Epitope binning of spike nanobodies.** Table S2**. Cryo-EM data collection, refinement and validation statistics. **Table S3**. Conservation of epitopic residues in RBD for C5G2 binding from natural occurring SARS-Cov-2 variants.

## Data Availability

Please contact the corresponding author for the data and materials.
